# Deficiency of complement receptors CR2/CR1 in *Cr2*^-/-^ mice reduces the extent of secondary brain damage after closed head injury

**DOI:** 10.1186/1742-2094-11-95

**Published:** 2014-05-24

**Authors:** Miriam D Neher, Megan C Rich, Chesleigh N Keene, Sebastian Weckbach, Ashley L Bolden, Justin T Losacco, Jenée Patane, Michael A Flierl, Liudmila Kulik, V Michael Holers, Philip F Stahel

**Affiliations:** 1 Department of Orthopedic Surgery, Denver Health Medical Center, University of Colorado School of Medicine, 777 Bannock Street, Denver CO 80204, USA; 2 Department of Medicine and Department of Immunology, University of Colorado, Campus Box B115, Barbara Davis Center, Aurora CO 80045, USA; 3 Department of Neurosurgery, Denver Health Medical Center, University of Colorado School of Medicine, 777 Bannock Street, Denver CO 80204, USA

**Keywords:** Closed head injury, Neuroinflammation, Complement receptor, *Cr2*^*-/-*^ mice, Secondary brain injury

## Abstract

Complement activation at the C3 convertase level has been associated with acute neuroinflammation and secondary brain injury after severe head trauma. The present study was designed to test the hypothesis that *Cr2*^*-/-*^ mice, which lack the receptors CR2/CD21 and CR1/CD35 for complement C3-derived activation fragments, are protected from adverse sequelae of experimental closed head injury. Adult wild-type mice and *Cr2*^*-/-*^ mice on a C57BL/6 genetic background were subjected to focal closed head injury using a standardized weight-drop device. Head-injured *Cr2*^*-/-*^ mice showed significantly improved neurological outcomes for up to 72 hours after trauma and a significantly decreased post-injury mortality when compared to wild-type mice. In addition, the *Cr2*^*-/-*^ genotype was associated with a decreased extent of neuronal cell death at seven days post-injury. Western blot analysis revealed that complement C3 levels were reduced in the injured brain hemispheres of *Cr2*^*-/-*^ mice, whereas plasma C3 levels remained unchanged, compared to wild-type mice. Finally, head-injured *Cr2*^*-/-*^ had an attenuated extent of post-injury C3 tissue deposition, decreased astrocytosis and microglial activation, and attenuated immunoglobulin M deposition in injured brains compared to wild-type mice. Targeting of these receptors for complement C3 fragments (CR2/CR1) may represent a promising future approach for therapeutic immunomodulation after traumatic brain injury.

## Introduction

Traumatic brain injury (TBI) is considered an ‘immunological disease’ due to the significant posttraumatic alterations in host-mediated immune functions in the central nervous system (CNS) and in the periphery [1-3]. The complement system has been implicated in the acute inflammatory pathophysiology of severe brain injury by mediating blood-brain barrier (BBB) breakdown, cerebral edema, and delayed neuronal cell death [4-6]. Complement C3 represents the central molecule of the complement cascade at the point where the three main initiation pathways (classical, alternative, and lectin) merge [7]. Elevated post-injury C3 levels were found in the intracranial compartment in experimental TBI models in rats, and in head-injured patients [8-10]. Both the depletion of C3 and the inhibition of C3 convertases has been shown to attenuate the extent of secondary brain injury in experimental animal models [11-13]. Complement receptor type 1 (CR1/CD35) and type 2 (CR2/CD21) are receptors for C3-derived activation fragments (C3b, iC3b, C3d, C3dg) [7]. Of these, CR2 has been identified as a crucial ‘link’ between innate and adaptive immunity by bridging the complement system to B cell-mediated humoral immune responses [14]. For example, CR2 forms an important co-receptor on B cells during antigen-induced activation through the B cell receptor (BCR) and subsequent signal transduction [7,14]. A recent focus of research has been devoted to the role of CR2 in the development and regulation of pathogenic natural antibodies that target neo-epitopes exposed in injured tissue [15]. Strikingly, mice lacking CR2 have been shown to be protected from the development of ischemia/reperfusion injury under a variety of experimental conditions [15,16]. In the mouse, the *Cr2* gene encodes both CR2 and CR1 receptors by alternative splicing [17]. The phenotype of genetically engineered *Cr2*^-/-^ mice deficient in CR2/CR1 is characterized by multiple immunological alterations, including an altered natural antibody repertoire and impaired B cell responses to antigen exposure [15,18]. In light of the important implications of the *Cr2* gene for innate and humoral immune responses, we hypothesized that *Cr2*^*-/-*^ mice are protected from secondary brain damage and adverse neurological outcome in a standardized model of experimental closed head injury [19].

## Materials and methods

### *Cr2*^*-/-*^ mice

The generation and characterization of *Cr2*^*-/-*^ mice has been previously described [18]. Breeding pairs for the mice used in the present study consisted of three male and two female homozygous *Cr2*^*-/-*^ mice that had been previously backcrossed to a pure C57BL/6 background. Breeding colonies were established at Denver Health Medical Center by crossing *Cr2*^*-/-*^ mice to wild-type littermates on a genetic C57BL/6 background (Jackson Laboratory, Bar Harbor, Maine, United States). The resulting heterozygous *Cr2*^*+/-*^ mice (F1) were then bred to each other to obtain 25% homozygous *Cr2*^*-/-*^ mice in the next generation (F2). Only homozygous *Cr2*^*-/-*^ mice were used for this study, and were identified by genotyping using real-time polymerase chain reaction (PCR) analysis of tail biopsies (Transnetyx, Cordova, Tennessee, United States). A total of n = 102 *Cr2*^*-/-*^ mice and n = 151 C57BL/6 wild-type mice were used for the experiments in this study.

### *Rag1*^*-/-*^ mice

In order to investigate the potential indirect mechanisms of CR2 that may be related to effects mediated by altered levels of pathogenic natural antibodies, an additional set of experiments was designed that used *Rag1*^*-/-*^ mice which lack mature B and T lymphocytes, and are therefore incapable of producing natural antibodies by B-1 cells [20]. The phenotype of *Rag1*^*-/-*^ mice is characterized by small lymphoid organs but not linked to any neuroanatomical, neurological, or behavioral abnormalities [20]. Adult male *Rag1*^*-/-*^ mice (n = 90) on a C57BL/6 background (Jackson Laboratory, Maine, United States) were used to investigate the role of reconstitution of selected pathogenic natural antibodies in our model of closed head injury.

### Closed-head injury model

All mice used in this study were exclusively of male gender (to avoid a bias regarding gender-related susceptibility to brain injury), between 10 and 12 weeks of age, weighing 25 to 35 g, and housed in single cages for at least 7 days before being subjected to experimental procedures. Mice were kept in a selective pathogen-free (SPF) environment under standardized conditions of temperature (21°C), humidity (60%), light and dark cycles (12 h:12 h), with food and water provided *ad libitum*. The study was performed in compliance with the National Institutes of Health (NIH) guidelines for the care and use of laboratory animals and was approved by the Institutional Animal Care and Use Committee of the University of Colorado (IACUC, protocol number B-79612(01)1E). A standardized weight-drop device was used for induction of focal closed head injury to the left brain hemisphere, as previously described [19]. In brief, after induction of isoflurane anaesthesia the skull was exposed by a longitudinal midline scalp incision. The head was fixed and a 333 g weight was dropped on the skull from a height of 2.5 cm, inducing a focal blunt injury to the left hemisphere. After trauma, all mice received supportive oxygenation with 100% O_2_ until fully awake. Analgesia was provided by an intraperitoneal injection of 0.05 mg/kg fentanyl immediately prior to the experimental procedure, followed by an injection of 0.01 mg/kg fentanyl every 12 hours until sacrificed. Sham-operated mice underwent identical procedures with regard to analgesia, anaesthesia, and surgical scalp incision, but were not subjected to experimental head trauma. For each set of experiments, four to five mice were used per time-point and condition. Cumulative numbers of mice per group were used for determining neurological outcomes for up to seven days after trauma.

### Neurological severity score (NSS)

The NSS represents a standardized scoring system for the quantification of post-injury neurological outcome, as described in detail elsewhere [19]. The score comprises 10 individual parameters including tasks on motor function, alertness, and physiological behavior. One point is awarded for the absence of a tested reflex or for the inability to perform a specific task, and no point for succeeding.

**Figure 1:**
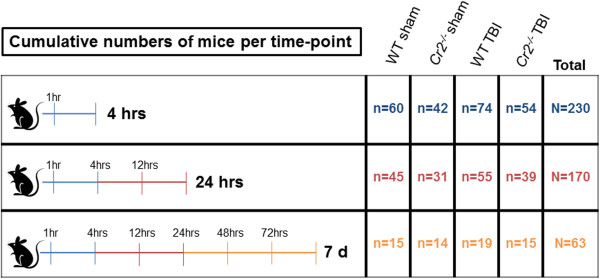
**Schematic depiction of cumulative numbers of mice (overall N = 230) that underwent neurological scoring, stratified by experimental groups.** d, days; TBI, traumatic brain injury; WT, wild-type.

A maximum NSS of 10 points indicates severe neurological dysfunction with failure of all tasks, whereas a low NSS of 0 to 2 points is characteristic of healthy uninjured mice [19]. In this study, the NSS was assessed at the time-points 1, 4, 12, 24, 48, and 72 hours, and 7 days after trauma or sham surgery by two investigators who were blinded to the study groups. The cumulative numbers of animals included in the NSS scoring (N = 230) and stratification by experimental groups is explained in Figure 1.

### Harvest of blood samples and brain tissue

Animals were sacrificed by decapitation under isoflurane anaesthesia at *t* = 4 hours, 24 hours, and 7 days after trauma or sham surgery. Whole blood was taken by intracardiac puncture immediately prior to decapitation. For collection of serum samples, whole blood was transferred to sterile serum microtubes (Sarstedt, Nümbrecht, Germany), centrifuged at 10,000 g for 10 minutes (4°C) and the supernatants were frozen at -80°C until analysis. For collection of plasma samples, whole blood was collected in sterile microtubes containing 3.5% ethylenediaminetetraacetic acid (EDTA) and a protease inhibitor cocktail (Sigma-Aldrich, St Louis, Missouri, United States). Samples were centrifuged at 2,000 g for 15 minutes (4°C) and the obtained plasma stored at -80°C until analysis. Brain samples were surgically removed after decapitation, snap-frozen in liquid nitrogen and stored at -80°C. Prior to analysis, brains were split into left (injured) and right (uninjured internal control) hemispheres, and the cerebellum was removed. For assessment of inflammatory and apoptotic mediators by western blots, brain hemispheres were homogenized with a Tissue Master-125 Homogenizer (Omni International, Kennesaw, Georgia, United States), as described in detail below.

### TUNEL histochemistry and NeuN double-immunostaining

A standard terminal deoxynucleotidyl transferase 2'-deoxyuridine-5'-triphosphate (dUTP) nick-end labeling (TUNEL) staining technique was used to determine the extent of neuronal cell death, using the *In Situ* Cell Death Detection Kit, TMR red (Roche Diagnostics, Mannheim, Germany). Sections were double-stained with a neuronal marker to differentiate TUNEL positive neurons from other cells types. In brief, frozen slides with serial coronal brain cryosections of 10 μm thickness were air-dried for 30 minutes, followed by fixation in 10% formalin solution (Sigma-Aldrich) for 10 minutes. After washing in phosphate-buffered saline (PBS), sections were blocked with a 3% normal goat serum (Jackson ImmunoResearch laboratories, West Grove, Pennsylvania, United States) for 1 hour at 4°C, followed by incubation in a mixture of primary antibody and NeuN at a dilution of 1:300 (Millipore, Billerica, Massachusetts, United States; catalogue number MAB377) overnight at 4°C. Sections were rinsed and incubated in a mixture of goat anti-mouse IgG-FITC secondary antibody at a dilution of 1:1000 (Abcam, Cambridge, Massachusetts, United States) for 2 hours at room temperature. Slides were washed again and permeabilized in 3% Triton X-100 solution (Sigma-Aldrich) for 60 minutes. Slides were then incubated with the terminal deoxynucleotidyl transferase (TdT) enzyme and tetramethylrhodamine (TMR)-labeled dUTP for 90 minutes at 37°C. Negative control sections were incubated in absence of the TdT enzyme. For positive controls, equal brain sections were exposed to 500 U/ml DNase grade I solution (Roche Diagnostics) for 20 minutes. Slides were then cover-slipped with Vectashield mounting medium containing 4’, 6’-diamino-2-phenylindole (DAPI; Vector Laboratories, Burlingame, California, United States). All sections were evaluated immediately after staining using an Olympus BX41 fluorescence microscope (Olympus, Center Valley, Pennsylvania, United States) at 525 nm for NeuN fluorescence (green) and at 576 nm for TUNEL TMR (red). Data were analyzed by QCapturePro7 software (QImaging, Surrey, British Columbia, Canada). The TUNEL-positive cells were counted in 15 randomly selected cortical fields of 0.01 mm^2^ per section. Only cells with a strong fluorescent signal for both red (TUNEL) and green fluorescence (NeuN) were counted. Cell counts were analyzed as mean values ± SD.

### Western blot analysis

Protein levels of complement C3, pro-apoptotic (Fas-L, Fas, Bax), and anti-apoptotic (Bcl-2) mediators were assessed in brain homogenates and plasma samples by western blotting. Separated brain hemispheres were homogenized with a Tissue Master-125 homogenizer (Omni International) in a radioimmunoprecipitation assay (RIPA) lysis buffer containing 12.1 mM sodium deoxycholate, 3.5 mM sodium dodecyl sulphate (SDS), 0.6 mM phenylmethanesulfonyl fluoride (PMSF), 1 mM sodium orthovanadate, 1% igepal CA-630 and 5% protease inhibitor cocktail (Sigma-Aldrich) in PBS. Homogenized samples were centrifuged at 16,000 g for 15 minutes (4°C) and the supernatants stored at -80°C. For western blot analysis, total protein concentrations of brain homogenates and plasma samples were determined using a colorimetric assay (BCA Protein Assay; Thermo Scientific, Rockford, Illinois, United States). Equivalent amounts of 50 μg protein were then denatured in loading buffer (Laemmli sample buffer + 5% mercaptoethanol) and separated under reducing conditions on 10% (Fas), 12.5% (C3, Fas-L and β-Actin) or 15% (Bax and Bcl-2) sodium dodecyl sulfate (SDS) polyacrylamide gels. Proteins were transferred to nitrocellulose membranes using a dry electroblotting iBlot system (Invitrogen, Carlsbad, California, United States). Membranes were then blocked with 5% non-fat milk (Nestle, Wilkes-Barre, Pennsylvania, United States) for 60 minutes and incubated overnight at 4°C with either polyclonal anti-Fas (Santa Cruz Biotechnology, Santa Cruz, California, United States; catalogue number Sc-1023), polyclonal anti-Fas ligand (Fas-L, Santa Cruz Biotechnology; catalogue number Sc-6237), monoclonal anti-Bcl-2 (Santa Cruz Biotechnology, catalogue number Sc-7382) or monoclonal anti-Bax antibodies (Santa Cruz Biotechnology, catalogue number Sc-80658), each diluted at a ratio of between 1:300 and 1:600, as appropriate. For detection of complement C3, a monoclonal anti-C3 antibody from Santa Cruz Biotechnology (catalogue number Sc-28294) was used for analysis of brain homogenates (diluted 1:10), and a monoclonal anti-C3 antibody clone ‘3d11’ from our own laboratory was used for analysis of plasma samples (diluted 1:1,000). To ascertain equal loading, membranes were incubated with a monoclonal anti-β-actin antibody from Santa Cruz Biotechnology (catalogue number Sc-47778), diluted 1:1,000. After incubation with alkaline phosphatase (AP)-conjugated secondary antibodies (Jackson ImmunoResearch) for 60 minutes (diluted 1:5,000), AP detection occurred in a nitro-blue tetrazolium/5-bromo-4-chloro-3'-indolyphosphate (NBT/BCIP) stock solution (Roche Diagnostics). The following whole cell lysates were used as internal positive controls: HL-60 for Fas-L; A-431 for Fas; CTLL-2 for Bax; and mouse spleen extract for Bcl-2 (all from Santa Cruz Biotechnology). Western blot band intensities were quantified using ImageJ software (Bethesda, Maryland, United States).

### Immunohistochemistry (IHC)

IHC was performed in serial coronal brain cryosections of 10 μm thickness using a standard biotin/avidin/peroxidase technique with diaminobenzidine (DAB) as chromogen (Vector Laboratories), as previously described [21,22]. The following primary antibodies and dilutions were used: monoclonal anti-mouse C3 (1:20) for assessment of C3 deposition in injured brain tissue (Hycult Biotech, Plymouth Meeting, Pennsylvania, United States; catalogue number HM1045); monoclonal anti-mouse NeuN (1:400) as a neuronal cell marker (Millipore; catalogue number MAB377); monoclonal anti-mouse glial fibrillary acidic protein (GFAP) (1:40) as a marker for astrocytes (Santa Cruz Biotechnologies; catalogue number Sc-33673); monoclonal anti-mouse CD11b (1:200) as a marker for microglia (AbD Serotec, Raleigh, North Carolina, United States; catalogue number MCA711); and polyclonal goat anti-mouse immunoglobulin M (IgM) (μ chain, Jackson ImmunoResearch; catalogue number 115-001-020) for detection of IgM deposition in brain tissue (1:100). All cryosections were counterstained with hematoxylin (Dako, Carpinteria, California, United States), cover-slipped and analyzed using an Olympus BX41 microscope and Altra 20 imaging software (Olympus). Positive IgM, microglia, and astrocytes were quantified using OCapturePro7 software (QImaging, Surrey, British Columbia, Canada). Positive IgMs were counted in five randomly selected fields of 0.01 mm^2^ per section. Positive CD11b and GFAP cells were tagged for intensity reading and classification of reactive microglia and astrocytes was determined based on location and signal intensity per 0.0625 mm^2^ of two different brain sections.

### ELISA

Serum samples were analyzed for mouse tumor necrosis factor (TNF), interleukin (IL) -6, and IL-10 using commercially available ELISA kits, according to the manufacturer’s instructions (R&D Systems, Minneapolis, Minnesota, United States). In addition, serum levels of neuron-specific enolase (NSE), an established marker of neuronal cell death after head injury, were determined by a commercially available ELISA, according to the manufacturer’s protocol (Immuno-Biological Laboratories, Minneapolis, Minnesota, United States). The C3-derived complement activation product C3a was assessed in plasma samples using a mouse anaphylatoxin C3a-specific ELISA developed in our laboratory, as previously described [21]. Optical density was read at 405 nm (NSE) or 450 nm (C3a and cytokines) using an Anthos 2010 plate reader (BioTek, Winooski, Vermont, United States).

### Purification and reconstitution of monoclonal natural IgM

Monoclonal IgM antibodies (mAbs) were developed by the fusion of peritoneal, lymph node, and spleen cells from wild-type C57BL/6 mice with the Sp2/0-Ag14 myeloma cell line to establish IgM-producing hybridomas, as previously described [23,24]. Successful fusions were confirmed by western blot analysis. All reagents used for the purification of mAbs were purchased from Sigma-Aldrich. Three positive hybridomas were selected for the present study: B4 (mAbs recognizing annexin IV); C2 (mAbs recognizing a subset of phospholipids); and F632 (isotype-matched control mAb). Supernatants of cultured hybridomas were affinity purified on columns coated with agarose beads and anti-human IgM (μ chain), which has been previously shown to be cross-reactive with mouse IgM [23]. Bound IgM antibodies were washed in PBS containing 0.02% sodium azide (pH 7.4), eluted with 0.2 M glycine (pH 2.3), and collected in a 1.5 M TRIS buffer (pH 8.8). Eluted antibody was concentrated by centrifugal filtration using Centricon Plus-70 centrifugal filter units (EMD Millipore). Final antibody concentration was measured by spectrophotometry at an excitation wave length of 280 nm. Antibody purity and specificity was confirmed by 10% SDS-gel electrophoresis. The concentrates containing mAbs B4 and C2 and the isotype control (mAb F632) were individually dissolved in sodium azide-free PBS and stored under sterile conditions at 4°C. Natural IgM (mAb B4 and mAb C2) and the isotype control mAb (F632) were administered by an intravenous injection into the animals’ penile vein, at an individual dose of 50 μg dissolved in 200 μl sterile PBS. Injections were performed immediately prior to the experimental condition (head injury or sham surgery), and repeated at *t* = 24 hours for analysis of samples at later time-points beyond 24 hours. The reconstitution of pathogenic natural antibodies occurred after the neurological scoring (NSS) at 24 hours. The selected dosage was determined by previous dose-response studies in *Rag1*^*-/-*^ mice, showing no difference in the extent of pathogenic effects at doses between 25 μg and 100 μg per mouse [23,24].

### Statistical analysis

Statistical analysis was performed using commercially available software (SigmaPlot 11.0, Systat Software, San Jose, California, United States). The repeated measures analysis of variance (ANOVA) was used to assess differences in NSS scores. Differences in post-injury mortality were evaluated on a contingency table using χ^2^ and Fisher’s exact test as appropriate. Differences in serum and plasma mediator levels determined by ELISA, as well as between cohorts assessed by semi-quantitative counts for TUNEL, GFAP, CD11b, and IgM, were evaluated by the unpaired Student’s *t*-test. *P* <0.05 was considered statistically significant.

## Results

### Neurological outcome

**Figure 2:**
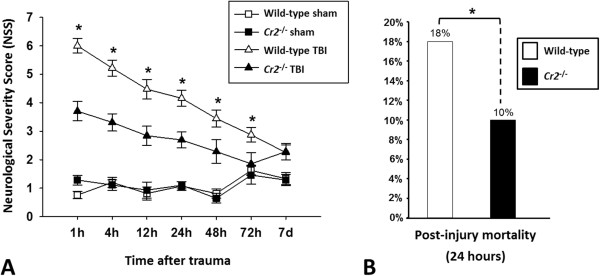
**Improved neurological outcomes and decreased post-injury mortality of *Cr2***^***-/- ***^**mice after traumatic brain injury (TBI). ***Cr2*^*-/- *^mice showed a significantly decreased Neurological Severity Score (NSS) from 1 hour to 72 hours after brain injury **(A)**, and a significantly decreased seven-day mortality **(B)**, compared to wild-type littermates. *P 0.05, repeated measures ANOVA (NSS); and χ^2^ test (mortality). Sham-operated animals served as internal controls. Data are shown as mean values ± SD.

Head-injured *Cr2*^*-/-*^ mice showed a significantly improved neurological outcome, as determined by a decreased NSS from 1 hour to 72 hours after trauma (*P* <0.05), compared to head-injured wild-type animals (Figure 2A). The NSS at seven days was in a similar range between head-injured *Cr2*^*-/-*^ mice (2.29 ± 0.29), and wild-type animals (2.25 ± 0.27), and in close proximity to the baseline NSS seen in sham-operated mice in both groups, which reflects the spontaneous neurological recovery at seven days after trauma in this model [19]. Finally, the post-injury mortality was significantly decreased in *Cr2*^*-/-*^ mice (10%), compared to wild-type mice (18%; *P* <0.05; Figure 2B).

### Neuronal cell death

**Figure 3:**
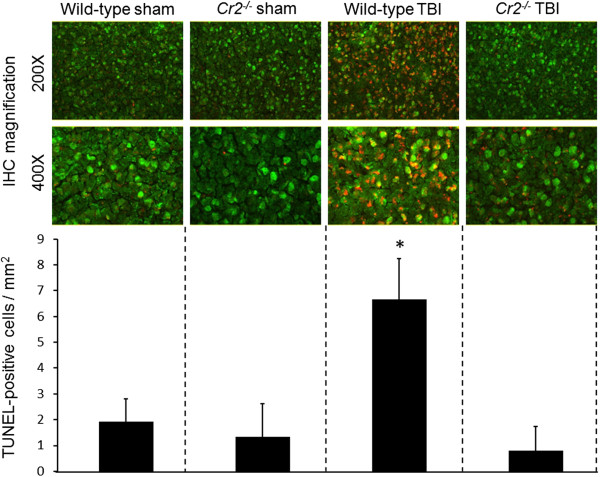
**Neuronal cell death is reduced in the injured brain hemispheres of *Cr2***^***-/- ***^**mice at seven days after traumatic brain injury (TBI).** Coronal cryosections of injured left hemispheres of wild-type and *Cr2*^*-/-*^ mice at seven days after sham operation or closed head injury were double-stained with a monoclonal anti-NeuN in green fluorescence (FITC) and by TUNEL technique in red fluorescence (TRITC), followed by visualization of cellular signals by fluorescence microscopy. Semi-quantitative analysis of cell-counts revealed a significantly increased number of TUNEL-positive neurons in the injured cortex of wild-type animals, compared to head-injured *Cr2*^*-/-*^ mice and sham-operated controls. The TUNEL-positive cells were counted in 15 randomly selected cortical fields of 0.01 mm^2^ per section. Cell counts are shown as mean values ± SD. *P <0.05 for wild-type TBI compared to *Cr2*^*-/-*^ TBI and sham controls. IHC, immunohistochemistry; TBI, traumatic brain injury.

TUNEL histochemistry was performed in coronal brain sections of *Cr2*^*-/-*^ and wild-type mice to assess the extent of intracerebral cell death after closed head injury. Very low baseline TUNEL fluorescence signals (red) were detected in the cortex of sham-operated mice in both groups (Figure 3). The mean cell count of TUNEL-positive cells increased dramatically in the injured cortex of wild-type mice at seven days post injury, compared to head-injured *Cr2*^*-/-*^ mice and sham-operated animals (*P* <0.05). Double-staining with anti-NeuN as a neuronal cell marker (in green fluorescence) revealed that neurons were the predominant TUNEL-positive cell type (Figure 3).

### Complement C3 release in injured brains

**Figure 4:**
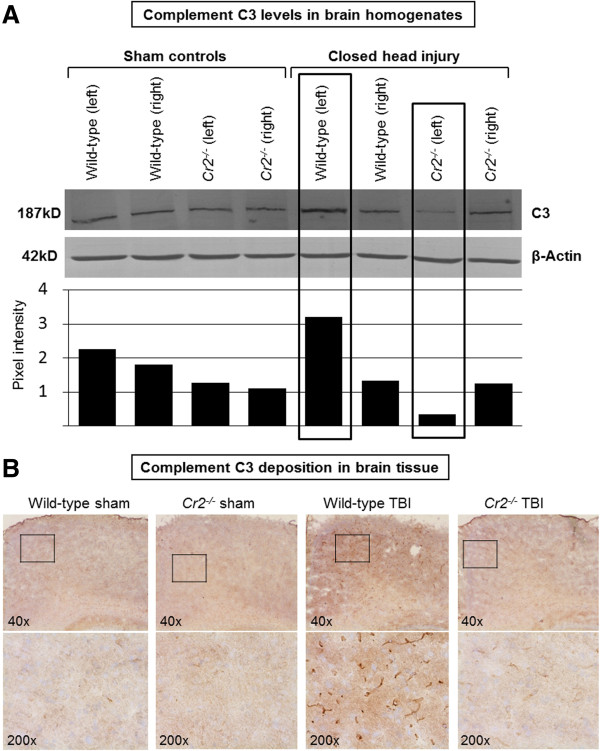
**Attenuated intracerebral complement C3 levels in head-injured *Cr2***^***-/-***^** mice at 24 hours post injury.** Panel **A** shows a representative western blot of brain homogenates from injured (left) and uninjured (right) hemispheres of wild-type and *Cr2*^*-/-*^ mice at 24 hours after head injury or sham surgery, using a monoclonal anti-mouse C3 antibody. Equal protein loading (50 μg per lane) was ensured by control blotting with β-actin. Closed head injury induced an increase of C3 protein levels in the injured hemispheres of wild-type mice compared to sham controls. In contrast, head-injured *Cr2*^*-/-*^ mice showed attenuated intracerebral C3 levels in the injured left hemisphere. Pixel intensity for densitometric quantification was determined using ImageJ software. The western blot shown in the figure is representative of four individual experiments. Panel **B** demonstrates the extent of C3 brain tissue deposition by immunohistochemistry using monoclonal anti-mouse C3 as primary antibody. Similarly as shown by the western blot data, C3 tissue deposition was attenuated in head-injured *Cr2*^*-/-*^ mice, compared to wild-type littermates. The inserts in the upper panel (40 ×) reflect the according selection in the corresponding higher magnification images (100 ×). TBI, traumatic brain injury.

Homogenized brain tissue and plasma samples were analyzed by western blotting for protein levels of the complement component C3. In analogy with findings from previous studies [8,10], C3 was upregulated in the brains of head-injured wild-type mice, compared to sham-operated controls, as shown in a representative western blot (Figure 4A). In contrast, brain homogenates from *Cr2*^*-/-*^ mice displayed a marked reduction of C3 protein levels after TBI, as quantified by densitometric analysis of the same representative blot shown in Figure 4A. Attenuated C3 levels in *Cr2*^*-/-*^ mice were specific to the injured left hemisphere, whereas the uninjured right (control) hemisphere showed similar C3 levels between the groups. In contrast to attenuated C3 levels in brain tissue from *Cr2*^*-/-*^ mice, western blot analysis of plasma samples revealed no difference in C3 levels between head-injured *Cr2*^*-/-*^ and wild-type mice, which implies that CR2/CR1-mediated effects related to binding of C3 fragments released after head injury are specific to the intracranial compartment. These findings are supported by IHC experiments which revealed an attenuated extent of C3 tissue deposition in injured brains of *Cr2*^*-/-*^ mice compared to wild-type animals (Figure 4B).

### Activation of astrocytes and microglia

**Figure 5:**
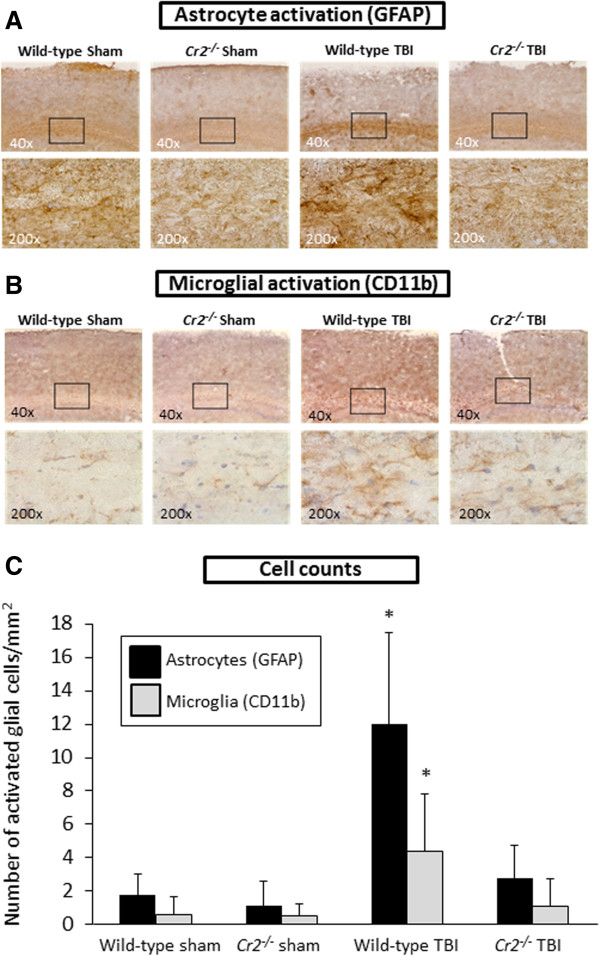
**Glial reaction is attenuated in *Cr2***^***-/-***^** mice at 24 hours after traumatic brain injury (TBI).** Immunohistochemistry of coronal brain cryosections was performed using glial fibrillary acidic protein (GFAP) as a specific marker for astrocytes **(A)** and CD11b as a cellular marker for microglia **(B)**. The inserts in the upper panel (40 ×) reflect the according selection in the corresponding higher magnification images (200 ×). Cell counts of immunostained sections revealed that signals for GFAP and CD11b were significantly increased in the injured left hemispheres of wild-type mice, compared to head-injured *Cr2*^*-/-*^ littermates **(C)**. Activated glial cells were counted in 10 randomly selected cortical fields of 0.01 mm^2^ per section and per cell marker. Cell counts were performed using QCapturePro7 software (QImaging).and values are shown as mean values ± SD. *P <0.05 for wild-type TBI compared to *Cr2*^*-/-*^ TBI and sham controls.

Cellular activation patterns of astrocytes and microglia were examined by IHC staining of serial coronal brain sections, using specific cell markers as primary antibodies (Figure 5). At 24 hours after TBI, brains from wild-type mice showed massive increase in staining intensity for GFAP-positive astrocytes (Figure 5A) and for CD11b-positive, ramified, microglia (Figure 5B) in the white matter of injured hemispheres. Cell counts of activated glial cells revealed that the intensity of cellular signals was significantly increased in head-injured wild-type animals compared to *Cr2*^*-/-*^ mice and sham controls (*P* <0.05; Figure 5C).

### Mediators of cell death and inflammation

**Figure 6:**
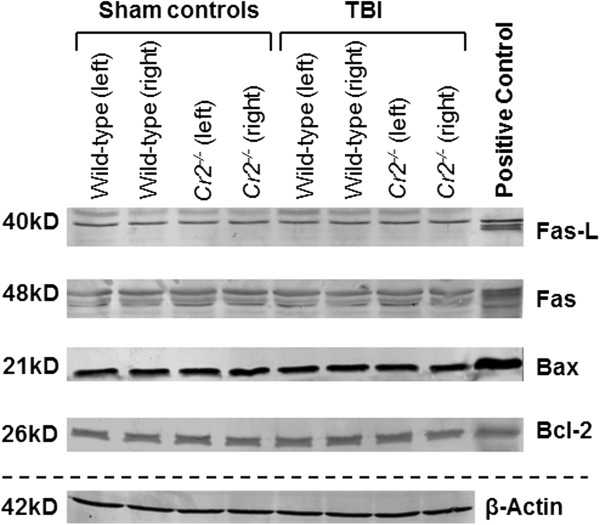
**Selected pro- (Fas, FasL, and Bax) and anti-apoptotic (Bcl-2) mediator levels in brain homogenates.** Samples were analyzed by western blots of injured (left) and non-injured (right) hemispheres at seven days after traumatic brain injury (TBI) or sham operation. Equal concentrations of protein (50 μg per lane) were loaded on SDS-Page, and consistent loading was confirmed by β-Actin control blotting. Mouse-specific primary antibodies against Fas, FasL, Bax, and Bcl-2 were used on nitrocellulose membranes and visualized by a colorimetric assay using alkaline-phosphatase. No differences in mediator levels were detected between the groups at any time-point assessed.

To investigate the potential mechanisms of attenuated neuronal cell death in head-injured *Cr2*^-/-^ mice, protein levels of selected apoptotic mediators in brain homogenates were assessed by western blot analysis. No differences in Fas, Fas-L, Bax, and Bcl-2 protein levels were observed at any time-point between head-injured *Cr2*^*-/-*^ compared to wild-type mice (Figure 6). These data imply that selected mediators of the extrinsic (Fas and Fas-L) and intrinsic (mitochondrial) pathway of apoptosis (Bax and Bcl-2) are not involved in the CR2/CR1-dependent regulation of neuronal cell death in our model of closed head injury in mice.

**Figure 7:**
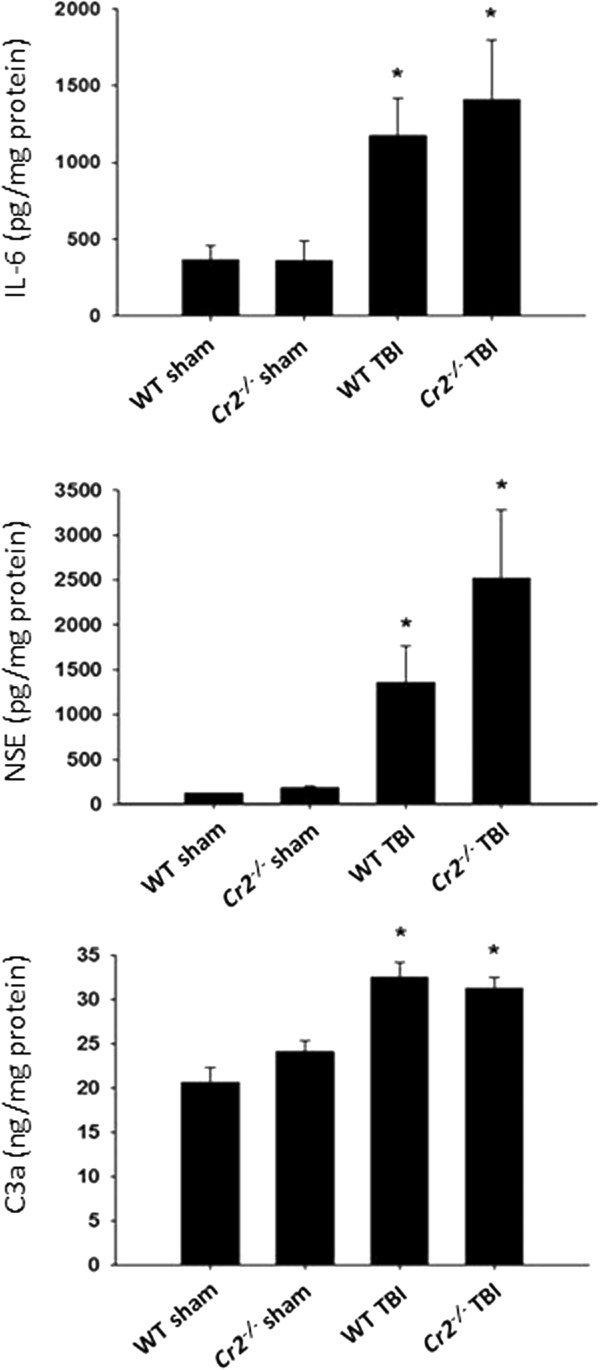
**Peripheral levels of selected biomarkers and inflammatory mediators in wild-type (WT) and *Cr2***^***-/-***^** mice at 24 hours after closed head injury or sham surgery.** Serum and plasma samples were analyzed by mouse-specific ELISA. Final mediator concentrations were normalized by total protein content of the samples, as described in the Materials and methods section. Head-injured mice showed a significant increase in mediator levels at 24 hours, compared to sham-injured animals (*P <0.05). However, there was no difference in concentrations between head-injured WT and *Cr2*^*-/-*^ mice. Data are shown as mean values ± SD and are representative of n = 22 WT and n = 13 *Cr2*^*-/-*^ mice.

For further investigation of potential underlying mechanisms of neuroprotection observed in head-injured *Cr2*^*-/-*^ mice, we investigated serum levels of NSE, as a surrogate marker of neuronal injury, selected pro- and anti-inflammatory cytokines (TNF, IL-6, and IL-10), and the plasma levels of the C3-derived anaphylatoxin C3a, by ELISA at *t* = 4 hours, 24 hours, and 7 days after trauma or sham surgery. Closed head injury induced a significant increase of serum IL-6 and NSE levels, as well as C3a plasma levels at 24 hours, in wild-type mice compared to sham-operated littermates (*P* <0.05; Figure 7). However, no significant differences in mediator levels were observed between head-injured *Cr2*^*-/-*^ and wild-type mice, at any time-point after trauma.

### Role of natural IgM

**Figure 8:**
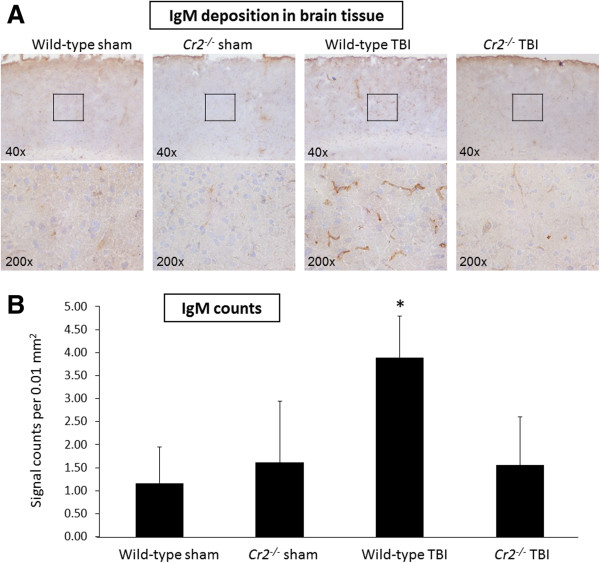
**IgM deposition is attenuated in *Cr2***^***-/-***^** mice at 24 hours after traumatic brain injury (TBI).** Immunohistochemistry of coronal brain cryosections was performed using an antibody specific to the μ chain for detection of IgM. Tissue deposition of IgM was markedly increased in the injured left hemispheres of wild-type mice. In contrast, head-injured *Cr2*^*-/-*^ mice showed low basal levels of IgM signals, to a similar extent as in sham-operated control mice **(A)**. The inserts in the upper panel (40 ×) reflect the according selection in the corresponding higher magnification images (200 ×). For quantitative analysis, IgM signals were counted in 20 randomly selected cortical fields of 0.01 mm^2^ per section **(B)**. Cell counts were performed using QCapturePro7 software (QImaging).and values are shown as mean values ± SD. *P <0.05 for wild-type TBI compared to *Cr2*^*-/-*^ TBI and sham controls. IgM, immunoglobulin M.

In light of the known protection of *Cr2*^*-/-*^ mice to tissue injury related to the lack of CR2-dependent signal transduction through the B cell receptor on B1 cells, one of the mechanisms of neuroprotection seen in head-injured *Cr2*^*-/-*^ mice may relate to the attenuated release of pathogenic natural IgM antibodies which target neo-epitopes on injured cells [15]. In order to determine whether head-injured *Cr2*^*-/-*^ mice may be protected through direct mechanisms related to the absence of CR2 and CR1, or indirectly by dysfunction of the CR2-dependent natural IgM repertoire, we performed additional IHC experiments to stain for IgM deposition in injured brains. Using antibodies specific for the immunoglobulin μ chain, low background staining was seen in sham-operated wild-type and *Cr2*^*-/-*^ mice sacrificed at 24 hours (Figure 8A). Head-injured wild-type mice showed a significant increase in IgM deposition at 24 hours after trauma by semi-quantitative signal count analysis (*P* <0.05; Figure 8B). In contrast, *Cr2*^*-/-*^ mice had a drastically attenuated IgM staining pattern in injured brains, which was similar to the baseline staining seen in sham-operated mice (Figure 8). These data suggest that indirect mechanisms related to a defective IgM repertoire in the absence of CR2 on B1 cells may be in part responsible for the neuroprotective features seen in head-injured *Cr2*^*-/-*^ mice.

**Figure 9:**
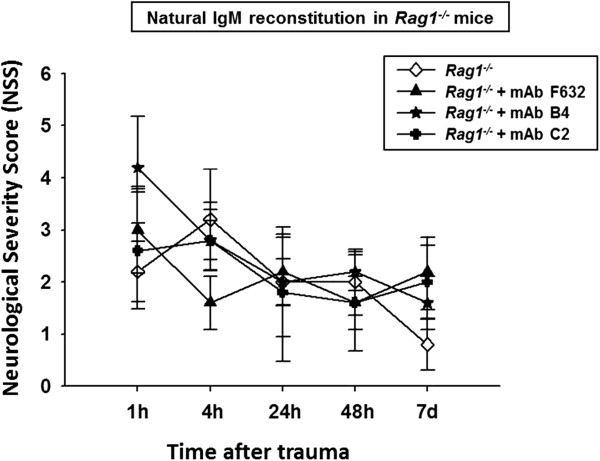
**Reconstitution of selected pathogenic natural antibodies in immunodeficient *Rag1***^***-/- ***^**mice.** The pre-injury reconstitution of mAbs against annexin IV (mAb B4) and phospholipids (mAb C2) did not alter the neurological outcome in head-injured *Rag1*^*-/-*^ mice which lack a natural antibody repertoire. The Neurological Severity Score (NSS) was assessed for up to seven days after closed head injury in *Rag1*^*-/-*^mice. No significant difference was seen in neurological outcomes between head-injured *Rag1*^*-/-*^ mice with or without reconstitution of pathogenic natural antibodies (mAb B4 and mAb C2) or mAb F632 as an isotype-matched control antibody (repeated measures ANOVA; *P* >0.05). IgM, immunoglobulin M.

In order to further elaborate on the potential role of pathogenic natural IgM in contributing to secondary brain damage after TBI, additional experiments were designed in *Rag1*^*-/-*^ mice which lack B cells and T cells, and therefore lack a natural antibody repertoire [20]. For this purpose, reconstitution experiments were performed with selected pathogenic natural antibodies against annexin IV (mAb B4) or phospholipids (mAb C2) which had been previously linked to the pathogenesis of secondary tissue injury in other experimental models, including ischemic stroke and intestinal ischemia/reperfusion injury [23,24]. Head-injured *Rag1*^*-/-*^ mice reconstituted with pathogenic natural IgM showed no difference in neurological outcomes (NSS) for up to seven days, compared to *Rag1*^*-/-*^ mice without reconstitution or with injection of non-pathogenic IgM (mAb F632), as negative control (Figure 9).

## Discussion

The present study was designed to evaluate the role of CR2 and CR1, which are mainly expressed by B lymphocytes, in contributing to the secondary neuropathological sequelae of TBI. As these receptors have been implicated in the immunopathology of tissue injury in a variety of inflammatory conditions in and outside of the CNS, we hypothesized that mice with an inactive *Cr2* gene would show some extent of neuroprotection in a standardized model of experimental closed head injury [19]. Indeed, head-injured *Cr2*^*-/-*^ mice displayed a significantly improved neurological outcome, significantly reduced posttraumatic mortality, attenuated extent of neuronal cell death and of post-injury astrogliosis, and reduced intracerebral complement C3 deposition compared to wild-type littermates. Complement C3 accumulation in the injured cortex has been previously described in a fluid-percussion TBI model in mice, and intracerebral C3d deposition was found in post-ischemic mouse brains following experimental ischemic stroke [8,24]. In addition, the CNS-targeted blockade of C3 convertases in GFAP-*Crry* transgenic mice resulted in significantly improved neurological outcomes for up to four weeks after closed head injury, based on the identical experimental model used in the present study [11]. Insights from pharmacological approaches of inhibiting C3 convertases in experimental TBI models support the notion of complement C3-derived downstream activation products playing a crucial contributory role in post-injury neuroinflammation and neurodegeneration [13,25]. In these studies, neuronal subsets in the hippocampal layers with the highest vulnerability to closed-head injury were protected by pharmacological inhibition of C3 convertases [13]. A more recent study confirmed this finding by demonstrating that the C3 activation fragment C3d inhibits adult hippocampal neurogenesis, and that this pathogenic effect is mitigated in *Cr2*^*-/-*^ mice [26]. Arguably, this striking finding provides a potential explanation for the improved neurological outcomes in head-injured *Cr2*^*-/-*^ mice seen in the present study (Figure 2). This notion is supported by the observation of decreased C3 deposition in injured brains from *Cr2*^*-/-*^ mice by IHC, and decreased C3 protein levels in brain homogenates by western blotting (Figure 4), implying a causative role of C3-derived complement activation fragment binding to CR2 and/or CR1 on these cells.

In addition to the proposed protective mechanism related to the lack of CR2 ligand binding (C3d, C3dg, and iC3b) in head-injured *Cr2*^*-/-*^ mice, the absence of CR2 and CR1 also appeared to protect from neuronal cell death in cortical neurons in injured brain hemispheres (Figure 3). We have previously shown that secondary neuronal cell death may be in part mediated by the terminal complement pathway through the membrane attack complex (MAC), and that the membrane-bound complement regulator CD59 protects from secondary cerebral insults and delayed neuronal cell death [22,27]. As one of the apparent mechanisms may be represented by programmed cell death through distinct apoptotic pathways, we sought to investigate the protein levels of selected apoptotic mediators of the extrinsic and intrinsic (mitochondrial) pathway of apoptosis in brain homogenates from *Cr2*^*-/-*^ and wild-type mice. Strikingly, no difference in protein levels of any of the selected pro- (Fas, FasL, and Bax) and anti-apoptotic (Bcl-2) mediators investigated in this study was seen between head-injured *Cr2*^*-/-*^ and wild-type mice, at any time-point investigated (Figure 6). These findings support the notion that mechanisms of protection from secondary neuronal cell death in the *Cr2*^*-/-*^ genotype are independent of apoptotic mechanisms and programmed cell death.

A different perspective of secondary host-mediated neuropathology relates to the crucial role of CR2 in the development and regulation of pathogenic natural antibodies targeting neo-epitopes expressed in injured tissue [15]. The *Cr2*^-/-^ phenotype is characterized by multiple immunological alterations, including an altered natural antibody repertoire and impaired B cell responses to antigen exposure [15,18]. In the present study we found a decreased extent of IgM deposition in the injured brains of *Cr2*^-/-^ mice compared to head-injured wild-type littermates (Figure 8). This finding is suggestive of a potential role of natural IgM, and of CR2-facilitated signal transduction and IgM release by B cells, in contributing to host-mediated secondary neuropathology after closed head injury. Natural antibodies are ‘hard-wired’ components of the immune system that circulate in absence of specific antigen exposure or immunization and exhibit several different types of functional attributes [15]. Some natural antibodies are low-affinity, polyreactive, non-immunized antibodies of the IgM isotype which are produced by a subset of B cells (termed B-1 cells) and represent a bridge between innate and adaptive immune responses [15,28]. Other natural antibodies have been shown to contribute to immunological homeostasis under physiological conditions and to the development of autoimmune disease [29]. In experimental studies, an additional subset of non-polyreactive pathogenic natural antibodies has been shown to mediate tissue damage in various models of ischemia/reperfusion injury and ischemic cerebrovascular stroke in mice [16,23,24]. The presumed mechanism manifested by these antibodies is the recognition of neo-antigens present on the surface of ischemic tissues, with subsequent pathogenic complement activation during the reperfusion phase, leading to unintentional host cell death [29-31]. In light of the impaired signal transduction in the absence of CR2 on B1 cells, and subsequently altered natural antibody response in *Cr2*^*-/-*^ mice, we designed an additional set of experiments aimed at elucidating the potential role of selected pathogenic natural IgM in contributing to secondary neuropathology in our model of closed head injury. For this purpose we used mice deficient in the *Rag1* gene (*Rag1*^*-/-*^) which are genetically devoid of mature B and T lymphocytes and therefore lack the complete natural antibody repertoire [20]. In contrast, the absence of CR2 expression by B lymphocytes in *Cr2*^*-/-*^ mice leads to an altered natural antibody repertoire, but these mice are still capable of producing natural IgM [15]. *Rag1*^*-/-*^ mice were reconstituted with two selected natural IgM against annexin IV and phospholipids, which have previously been shown to exert pathogenic effects in other mouse models of inflammation-mediated tissue injury (mAb B4 and mAb C2) [23,24,32]. Strikingly, we found that the pre-injury reconstitution of either mAb B4 or mAb C2 in immunocompromised *Rag1*^*-/-*^ mice does not alter the neurological outcome in comparison to head-injured *Rag1*^*-/-*^ mice injected with a non-pathogenic control IgM (mAb F632), or to *Rag1*^*-/-*^ mice without IgM reconstitution (Figure 9). In addition, the extent of complement activation at the C3 level and of complement C3 deposition in injured brain tissue was not altered in head-injured *Rag1*^*-/-*^ mice with or without IgM reconstitution (data not shown). Finally, neither the extent of post-injury neuronal cell death, nor the levels of selected mediators of cell death and inflammation were altered by reconstitution of natural antibodies in *Rag1*^*-/-*^ mice (data nor shown). These findings suggest that the two selected natural IgM investigated in this study (mAb B4 and mAb C2) do not exert pathogenic properties which contribute to the evolution of secondary brain injury in this model of closed head injury in mice.

The exact cellular and molecular mechanisms of CR2-mediated neuropathology seen in the present study remain speculative. Conceivably, the homeostasis between protective and harmful natural antibodies may be of crucial importance and outweigh the isolated pathogenic effects of the two selected natural IgM investigated in this study [33]. Similarly, the underlying root cause of the impressive attenuation of the extent of post-injury glial activation observed in head-injured *Cr2*^-/-^ mice in this study (Figure 5) remains a topic of speculation. Recent studies have emphasized an important role for activated microglia and reactive astrocytosis in contributing to post-injury neuroinflammation and adverse outcomes [34-36]. A recently published experimental study using the controlled cortical impact (CCI) model in Wistar rats revealed that microglial activation after focal TBI peaked within the first three to seven days post injury which reflects a similar timeline of cellular activation observed in the present study [34].

Impressively, a landmark article by Gasque *et al*. reported the expression of complement C3 receptors (CR1, CR2, and CR3) by human astrocytes *in vitro*[37]. The CR2 expressed on astrocytes was shown to be functional, as demonstrated by Epstein-Barr virus infection through the CR2 expressed in human astrocyte cell lines [38]. In a different study on a rat model of TBI, Bellander *et al.* were able to correlate enhanced microglial activation with intracerebral complement C3 expression at seven days post injury [9], which reflects the same time-window analyzed in our current study. Taken together, these data support the notion that complement C3 contributes to increased astrocytosis and microglial activation after TBI, likely through the CR2 expressed on astrocytes, and through indirect mechanisms on microglia since the latter cell-type does not express CR2 [9,37]. Our current findings of attenuated post-injury glial reaction in absence of the *Cr2* gene support this hypothesis (Figure 5). However, a potential role of CR1 in contributing to the post-injury neuropathology observed in our TBI model cannot be excluded, as the mouse *Cr2* gene encodes both CR2 and CR1 receptors by alternative splicing and *Cr2*^-/-^ mice are therefore deficient in both CR2 and CR1 [17,18].

**Figure 10:**
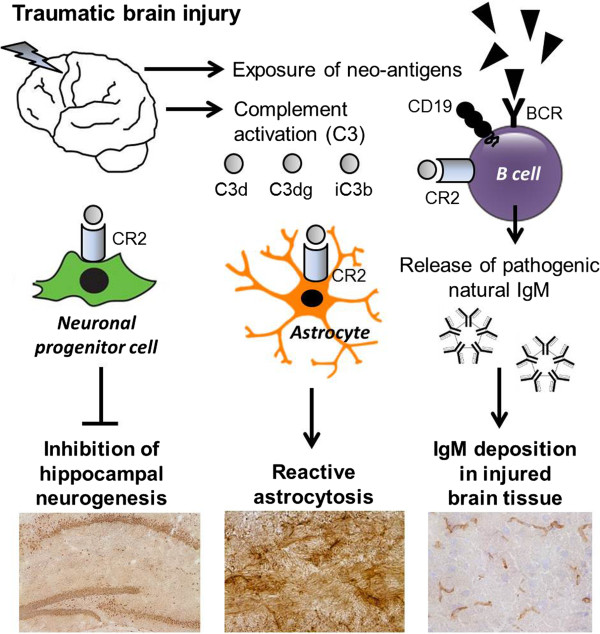
**Presumed mechanisms of CR2-mediated secondary neuropathology after closed head injury.** See discussion for detailed explanations. BCR, B cell receptor; CR2, complement receptor type 2.

Our current understanding of the potential underlying mechanisms of CR2-mediated neuropathology after TBI is depicted in a schematic diagram shown in Figure 10. In light of the recent availability of a new generation of recombinant chimeric compounds which link CR2 to inhibitors of the complement system, future studies should be designed to investigate the potential benefit of pharmacological modulation of CR2-mediated functions and of CR2-linked targeting of C3-derived complement fragments at the site of tissue injury [39].

## Abbreviations

AP: Alkaline phosphatase; BCR: B cell receptor; BBB: Blood-brain barrier; CNS: Central nervous system; CR1/CD35: Complement receptor type 1; CR2/CD21: Complement receptor type 2; DAB: Diaminobenzidine; DAPI: 4’, 6’-diamino-2-phenylindole; dUTP: 2'-deoxyuridine-5'-triphosphate; EDTA: Ethylenediaminetetraacetic acid; GFAP: Glial fibrillary acidic protein; IgM: Immunoglobulin M; IHC: Immunohistochemistry; IACUC: Institutional Animal Care and Use Committee of the University of Colorado; IL -6: Interleukin-6; IL-10: Interleukin-10; mAb: Monoclonal antibody; NIH: National Institutes of Health; NSS: Neurological severity score; NSE: Neuron-specific enolase; NBT/BCIP: Nitro-blue tetrazolium/5-bromo-4-chloro-3'-indolyphosphate; PBS: Phosphate-buffered saline; PCR: Polymerase chain reaction; SPF: Selective pathogen-free; SDS: Sodium dodecyl sulfate; TdT: Terminal deoxynucleotidyl transferase; TUNEL: Terminal deoxynucleotidyl transferase dUTP nick-end labeling; TMR: Tetramethylrhodamine; TBI: Traumatic brain injury; TNF: Tumor necrosis factor; WT: Wild-type.

## Competing interests

The authors declare that they have no competing interests.

## Authors’ contributions

PFS, MAF, and VMH designed this experimental study. MDN, MCR, CNK, JP, and SW performed the experimental animal procedures and assessment of all outcome parameters and tissue analyses. ALB, JTL, and SW designed and supervised the colony breeding process of the *Cr2*^*-/-*^ mice. LK provided the monoclonal anti-C3 antibodies used in this study. MAF and SW developed the anti-mouse C3a ELISA. MDN, MCR, and CNK provided the first draft of this manuscript. PFS and VMH revised the manuscript to its final draft. LK and VMH contributed with important discussion and interpretation of the results. All authors read and approved the final version of this manuscript.
